# Two is a Crowd: Two is a Crowd: On the Enigmatic Etiopathogenesis of Conjoined Twinning

**DOI:** 10.1002/ca.23387

**Published:** 2019-04-29

**Authors:** Lucas L. Boer, Annelieke N. Schepens‐Franke, Roelof Jan Oostra

**Affiliations:** ^1^ Department of Anatomy and Museum for Anatomy and Pathology Radboud University Medical Center Nijmegen The Netherlands; ^2^ Department of Medical Biology, Section Clinical Anatomy & Embryology, Academic Medical Center University of Amsterdam Amsterdam The Netherlands

**Keywords:** conjoined twins, fission, fusion, neo‐axial orientation, interaction aplasia, omphalopagus, thoracoileopagus, cephalothoracoileopagus, ileoischiopagus, parapagus, craniopagus, rachipagus, pygopagus, duplication

## Abstract

In this article, we provide a comprehensive overview of multiple facets in the puzzling genesis of symmetrical conjoined twins. The etiopathogenesis of conjoined twins remains matter for ongoing debate and is currently cited—in virtually every paper on conjoined twins—as partial fission or secondary fusion. Both theories could potentially be extrapolated from embryological adjustments exclusively seen in conjoined twins. Adoption of these, seemingly factual, theoretical proposals has (unconsciously) resulted in crystallized patterns of verbal and graphic representations concerning the enigmatic genesis of conjoined twins. Critical evaluation on their plausibility and solidity remains however largely absent. As it appears, both the fission and fusion theories cannot be applied to the full range of conjunction possibilities and thus remain matter for persistent inconclusiveness. We propose that initial duplication of axially located morphogenetic potent primordia could be the initiating factor in the genesis of ventrally, laterally, and caudally conjoined twins. The mutual position of two primordia results in neo‐axial orientation and/or interaction aplasia. Both these embryological adjustments result in conjunction patterns that may seemingly appear as being caused by fission or fusion. However, as we will substantiate, neither fission nor fusion are the cause of most conjoined twinning types; rather what is interpreted as fission or fusion is actually the result of the twinning process itself. Furthermore, we will discuss the currently held views on the origin of conjoined twins and its commonly assumed etiological correlation with monozygotic twinning. Finally, considerations are presented which indicate that the dorsal conjunction group is etiologically and pathogenetically different from other symmetric conjoined twins. This leads us to propose that dorsally united twins could actually be caused by secondary fusion of two initially separate monozygotic twins. An additional reason for the ongoing etiopathogenetic debate on the genesis of conjoined twins is because different types of conjoined twins are classically placed in one overarching receptacle, which has hindered the quest for answers. Clin. Anat. 32:722–741, 2019. © 2019 Wiley Periodicals, Inc.

## INTRODUCTION

The presence of ancient depicted cave drawings, carved figurines, and ceramics of human conjoined twins, as well as their opulent occurrence in the animal kingdom, strongly suggests that these malformations existed long before the human race finished descending from its ancestors (Berrin and Larco, 1997; Canfield et al., 2000; Pachajoa et al., 2014). Initially, the birth of a conjoined twin was seen as an inauspicious sign of impending disaster (Millar et al., 2009). This superstition‐filled era was followed by a prolonged period through the Middle Ages and well into the 19th century when conjoined twins were regarded as freaks or monstrosities and were exhibited at circuses and sideshows with substantial financial reward (Spitz, 2005). Conjoined twins rarely survive early infancy—approximately 30% dies in utero, 40%–60% are stillborn, and 35% survives 1 day (Casale et al., 2004; Millar et al., 2009). It is therefore that the birth and subsequent survival of a conjoined twin is matter for worldwide news, as they are seen as wonders and marvels of nature's creation (Kokcu et al., 2007) or even perceived as incarnations of deities (Tubbs et al., 2015). This wonderment on conjoined twins, which exists up to the present day, is of all times and all communities—eliciting strong emotions that vary between admiration to (maternal) rejection, repellence, and hostility or even infanticide (Bondeson, 1992; Mayer, 2001; DeSesso, 2019).

Early academic interest in gross teratological conditions—including conjoined twins—predominantly flourished in Europe between the 18th and early 20th century: the heydays of descriptive teratology (Beckwith, 2002). Between these pinnacles, prospectors who thought about possible etiopathogenetic causes were hampered by a lack of early (molecular) embryological knowledge, especially regarding the processes of (in)complete twinning. However, throughout multiple centuries, the etiopathogenesis of conjoined twins has crystallized into two currently conjectured theories: partial fission (Kaufman, 2004) versus secondary fusion (Spencer, 2003). Although both theories are postulated throughout literature, controversies remain existing (Mutchinick et al., 2011). Both the fission and fusion theories have crystallized in patterns of paraphrased and graphic representations in virtually every paper concerning conjoined twins (Mian et al., 2017). However, both theories show limitations, have overlapping dogmas and parlance—creating a potential susceptible situation for semantic interpretations. Most notably, the hypothetical deductions of these theories have (unconsciously) transited to allegedly verified factual embryological or (dys)morphological descriptions. An epistemic evolution changing an initial and tentative explanation or conceptualization into an accepted and undisputed theory is currently being observed. These “truths” should be critically reviewed and evaluated on their plausibility and (seeming) solidity.

In this article, we provide a comprehensive overview of multiple facets in the puzzling genesis of symmetrical conjoined twins. We will discuss the currently held etiopathogenetic views on the origin of conjoined twins and its commonly assumed etiological correlation with monozygotic twinning. In addition, arguments are given which indicate that the dorsal conjunction group is etiologically and pathogenetically different in comparison to other symmetric conjoined twins and could be caused by secondary fusion.

## FORMATION OF EARLY EMBRYONIC ORGANIZERS

Due to the different approach in the possible genesis of conjoined twins presented in this article, we feel the necessity to first highlight some basic considerations on the formation of early embryonic organizers. One highly regulative cell lineage during embryogenesis is the hypoblast (the anterior visceral endoderm [AVE] in the mouse) which controls epiblastic cell movements, ultimately leading to primitive streak formation and bilateral symmetry (Bellairs, 1953). In normal development, an isolated central epiblast disk cannot generate axial structures in the absence of the hypoblast (Azar and Eyal‐Giladi, 1981). It has to be noted that particularly in ducks, about 2% of the fertilized eggs form conjoined twins (Ulshafer and Clavert, 1979). The high prevalence of conjoined twins is assumed to be caused by orientation changes of the egg during critical periods of symmetrization. These movements change direction of rotation subsequently resulting in the formation of two organizing centers (Clavert, 1962). Waddington (1933) found that 90° rotation of the hypoblast before gastrulation in birds causes the orientation of the primitive streak to bend in the direction of the rotated hypoblast. The hypoblast transiently induces expression of preneural markers in the epiblast which contributes to delayed streak formation (Stern and Downs, 2012). In the mouse, the AVE is essential for the correct positioning of the primitive streak: AVE imparts anteroposterior polarity and potency on the primitive streak (Rodriguez and Downs, 2017). In knockout mouse embryos where AVE cells arrest or fail to be induced, the primitive streak is ectopic or even duplicated; highlighting the pivotal role of the AVE in streak positioning and formation (Bertocchini and Stern, 2002; Stower and Srinivas, 2014).

Besides the hypoblast, another major structure in embryogenesis is the primitive streak which plays a key role in the formation of the axial and paraxial mesoderm and the definitive endoderm from the epiblast (Spemann and Mangold, 1924). This structure will subsequently establish the whole fate map for ensuing embryological development initiating its ultimate morphology (Seleiro et al., 1996). The highly regulated primitive streak formation relies on a critical and concatenated, network of mainly three signaling activities at both transcriptional and signaling levels: BMP4 signaling activates the Wnt pathway which in turn activates the Activin–Nodal pathway (Ben‐Haim et al., 2006; Martyn et al., 2018). Activation of secretion factors like Vg1, nodal, Wnt8C, FGF8, and chordin completed with transcription factors such as brachyury and goosecoid adjacent to the site of streak establishment is required for streak formation (Skromne and Stern, 2002). In addition, during gastrulation, the left–right asymmetry is established by complex genetic signaling pathways and cilia‐mediated preferential flow at Hensen's node. This culminates in the exclusively left‐sided expression of the *NODAL* gene—which is mediated by SHH—in the lateral plate mesoderm (Sutherland and Ware, 2009).

## TYPES OF CONJOINED TWINS

The first discrimination in conjoined twins is the fact that some are symmetrical and others are not. The latter are characterized by gross underdevelopment of one of the twin members, commonly known as “parasites” or “heteropagi” (Sharma et al., 2010). We have chosen to exclude parasitic twins from the present discussion due to their complex and possibly heterogeneous nature.

The most commonly used classification divides symmetric conjoined twins into four general conjunction groups: ventral, lateral, caudal, and dorsal conjunction. In these four groups, 11 more or less well‐defined entities can be discriminated (Spencer, 2003). However many conjunction types show overlapping lateroventral, laterocaudal, and intermediate conjunction patterns, ultimately creating a divergent variability and heterogeneous phenotypical spectrum of conjunction; indicating a continuum between the different types of twins (Oostra et al., 1998).

### Nondorsal Conjunction

**Figure 1:**
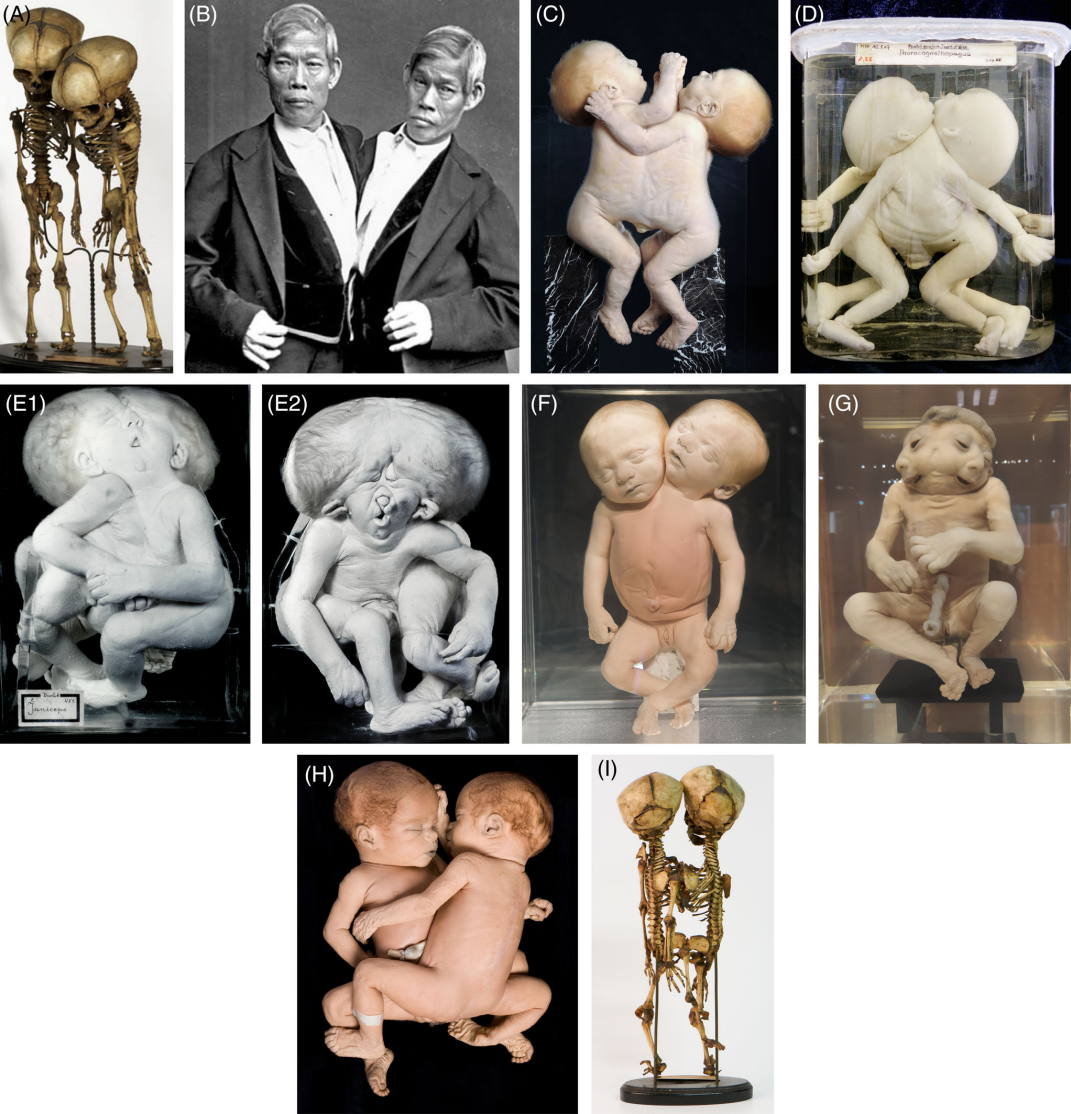
Nondorsally united twins. A, Skeleton of a xipho‐omphalopagus twins united at the mid‐ventral portion of the trunk. Specimen from the Vrolik Collection in Amsterdam (The Netherlands). B, Photograph of perhaps the most famous conjoined twins: Chang end Eng Bunker (1811–1974) born in Siam (Thailand) and the reason why the expression “Siamese twins” was coined. Chang and Eng were omphalopagi twins united in the epigastric region and mid‐abdominal area. C, Thoracoileopagus twins in which union starts mid‐sternally and extends to the umbilicus. Specimen from the Anatomical Museum in Nijmegen (The Netherlands). D, Prosopothoracoileopagus twins united ventrally from the face and/or neck to the umbilicus; the lower abdomen, genitalia, vertebral columns, limbs, and face are individually owned by each twin. Specimen from the Narrenturm collection in Wien (Austria). E, Cephalothoracoileopagus twins (left is “ventral” view, right is “dorsal” view) united throughout the entire head, two (complete) faces on opposite sides of a single conjoined head are noticeable. Specimen from the Vrolik Collection in Amsterdam (The Netherlands). F, Parapagus dicephalus twins with two heads on a single compound body. Specimen from the Anatomical Museum in Nijmegen (The Netherlands). G, Parapagus diprosopus twin with two laterally oriented faces in one compound head. Specimen from the Anatomical Museum in Nijmegen (The Netherlands). H, Ileoischiopagus tetrapus twins joined at the periumbilical and pelvic region—sharing the lower abdomen, pelvis, and perineum. Specimen from the Anatomical Museum in Nijmegen (The Netherlands). I, Skeleton of a thoracoileoischiopagus tribrachius tripus. Specimen from the Vrolik Collection in Amsterdam (The Netherlands). Note that all nondorsally united twins always have a single umbilicus and that vast amounts of the general body plan altered dramatically. [Color figure can be viewed at http://wileyonlinelibrary.com]

Classically, nondorsally conjoined twins can be divided into ventral, lateral, and caudal conjunction types. Ventrally conjoined twins are joined at the periumbilical regions and, with increasing degrees of union, the thorax, neck, face, and/or head can be additionally involved. A gradual spectrum exists between the different forms of ventral union. The mildest form of ventral conjunction is xipho‐omphalopagus (Fig. 1A) characterized by joined livers and a common peritoneal cavity (Lai et al., 1997). Xipho‐omphalopagi are successful candidates for surgical separation (Shukla et al., 2010). When conjunction becomes more profound, omphalopagus arises (Fig. 1B). The liver and diaphragm are involved in the union which can be additionally complicated by pericardiac and cardiac displacements—although no cardiac conjunction is present (McHugh et al., 2006). In approximately one‐third of the omphalopagi, shared intestines are seen (Winkler et al., 2008). Omphalopagi could be considered as candidates for surgical separation as well (Patil et al., 2016). Thoracoileopagus (Fig. 1C) twins show the same union as omphalopagi, but they share a single complex and composite heart with equal contributions from both twins (Spitz, 2005). Besides a compound heart, the liver, diaphragm, and proximal intestines are joined (Spencer, 2003). Thoracoileopagi are hardly ever separable because of the cardiac involvement (Winkler et al., 2008). Prosopothoracoileopagi (Fig. 1D) are united ventrally from the face and/or neck to the umbilicus. At the extreme end of the ventral union spectrum is cephalothoracoileopagus (Fig. 1E). These twins are united throughout the entire head, presenting with two (complete) compound faces on opposite sides of a single conjoined head; each twin contributing half of all conjoined structures. Both prosopothoracoileopagi and cephalothoracoileopagi are nonviable due to the often complex cardiovascular nature and the intricate degree of union, although the central nervous systems are individually owned by each twin.

Lateral conjoined twins are characterized by conjunction at the lateral aspects of the abdomen, thorax, neck, face, and/or heads and classically consist of two gradually overlapping entities: parapagus dicephalus with two heads (Fig. 1F) and parapagus diprosopus with two laterally oriented faces in one compound head (Fig. 1G). In contrast to the former entities, laterally conjoined twins share vast parts of their body. In addition, dicephalic twins can be further divided into dicephalus tetrabrachius (four arms), tribrachius (three arms) and dibrachius (two arms). Finally, the affix dipus and tripus can be included in the nomenclature to describe the amount of the present lower limbs. The extensive union in both dicephali and diprosopi usually precludes the possibility of separation and most die in the perinatal period (Winkler et al., 2008).

The third entity in the spectrum of nondorsally united twins is the caudally conjoined twins called ileoischiopagus (Fig. 1H). These twins are joined at the periumbilical and pelvic region—sharing the lower abdomen, pelvis, and perineum. Classically, two vertebral columns are located in a 180° opposite position. Twins may be oriented face to face, creating considerable variation in the angle between the two spines ranging from 15° to 180°. Ileoischiopagi usually share four upper and four lower limbs and two separate hearts (Spencer, 2003). Assessment of the pelvic osteology, genitourinary system, lower intestine, and rectum, and the degree of vascular sharing are the most important considerations for separation (Winkler et al., 2008).

Noteworthy is that all nondorsally conjoined twins share the periumbilical region and therefore have a single umbilical cord, which is sometimes flanked by a single overarching omphalocele (Lai et al., 1997). It is this premise, together with the often encountered intermediate conjunction patterns (Fig. 1I), which converge the ventrally, laterally, and caudally united twins into one overlapping phenotypical spectrum. In addition, this spectrum is affected by two embryological adjustments—exclusively seen in the nondorsally conjoined group (see further).

### Dorsal Conjunction

Classically, three types of dorsally united twins are discriminated. Noteworthy is that all dorsally united twins show individual internal organs and two separate umbilical cords; it is this peculiarity that discriminates the dorsally conjoined group from the nondorsally united twins.

**Figure 2:**
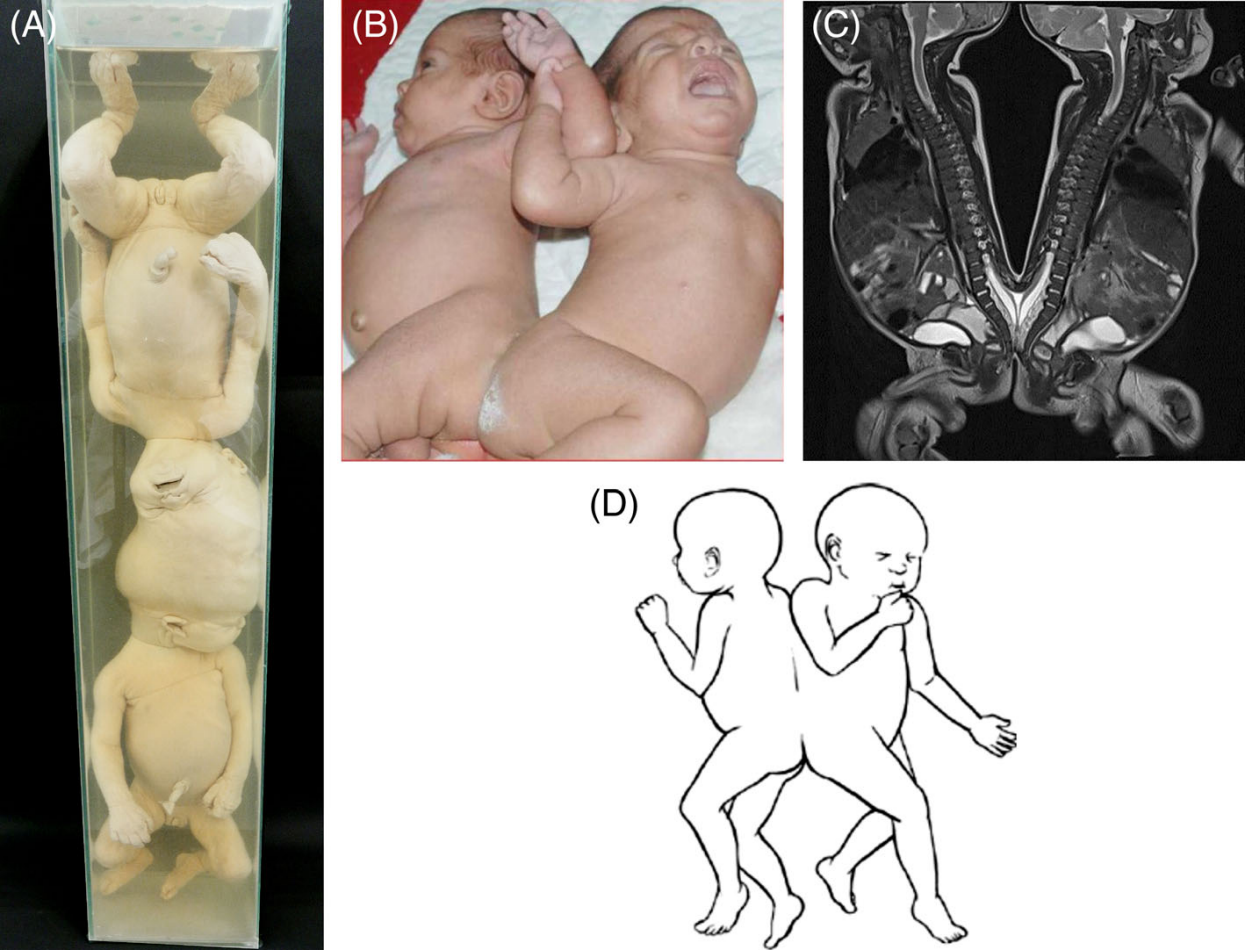
Dorsally united twins. A, Craniopagus twins with nonhomologous union at the head. Specimen from the Narrenturm collection in Wien (Austria). B, Pygopagus twins joined at the sacrum, coccyx, and perineum, facing away from each other (Awasthi et al., 2015). C, MRI of the child depicted in (B) which revealed a spina bifida from the fourth lumbar vertebra downward and low‐lying spinal cords tethered at the fifth lumbar vertebra to the first sacral vertebra. The filum terminale was fused at the second and third sacral vertebra within a single thecal sac. D. Drawing of rachipagus twins, united at the spine and facing away from each other. Note that all dorsally united twins always have two separate umbilical cords, individual internal organs, and lack in gross underdeveloped regions or dysmorphologies, which are almost invariably present in nondorsally united twins. B and C: Reused with permission from Awasthi R, Iyengar R, Rege S, Jain N, Eur Spine J, 2015, 24 Suppl 4, S560–S563, Springer Nature. D: Adapted from Spencer R, Conjoined Twins: Developmental Malformations and Clinical Implications, 2003, JHU Press. [Color figure can be viewed at http://wileyonlinelibrary.com]

Craniopagus twins are joined at the head, more specifically the cranial vault (Fig. 2A). The site of cranial union can be subdivided into frontal, temporal, parietal, or occipital union, but infinite variations exist in both axial and rotational orientation, ultimately leading to heterogenic phenotypes with marked nonhomologous connections such as frontoparietal, temporoparietal, and occipitoparietal union (Spencer, 2003). The juncture may include the meninges, the superior sagittal sinus, and, in some cases, show cerebral deformities and conjoined brain tissue—the latter often shows separable leptomeninges overlying the interdigitated gyri (O'Connell, 1976; Stone and Goodrich, 2006). Generally, it can be stated that the more extensive the union, the greater the decrease of relative calvarial volume and the greater the reciprocal pressure on the two developing brains (O'Connell, 1976). Union never involves the foramen magnum, the base of the skull, face, and vertebrae; the latter are however involved in cranio‐rachipagus twins (see further). The most important considerations for separation of craniopagi are the intricacy of shared dural venous sinuses and the amount of conjoined brain tissue (Winkler et al., 2008).

Pygopagus twins are united at the sacrum, coccyx, and perineum (Fig. 2B). Union often involves the dural sheath and the terminal portion of the spinal cord (Spencer, 2003). Structures originating from the secondary neurulation (e.g., the conus medullaris and filum terminale), which arise after the closure of the caudal neuropore, are often communal in pygopagi (Fieggen et al., 2003). The degree of dural and spinal cord conjunction and perineal, genitourinary, and sacrococcygeal morphology are the most important considerations for a possible separation (O'Connell, 1976).

Rachipagus twins are conjoined at the back (Fig. 2C). Only two reports of nonparasitic rachipagi exist, described by Bétoulières et al. (1960) and Durin et al. (2005). Both cases concern rachipagi with cranial conjunction (cranio‐rachipagus). This entity belongs to one of the rarest forms of conjoined twinning. Union may include the entire vertebral columns and occipital regions.

## EMBRYOLOGICAL ADJUSTMENTS IN CONJOINED TWINS

**Figure 3:**
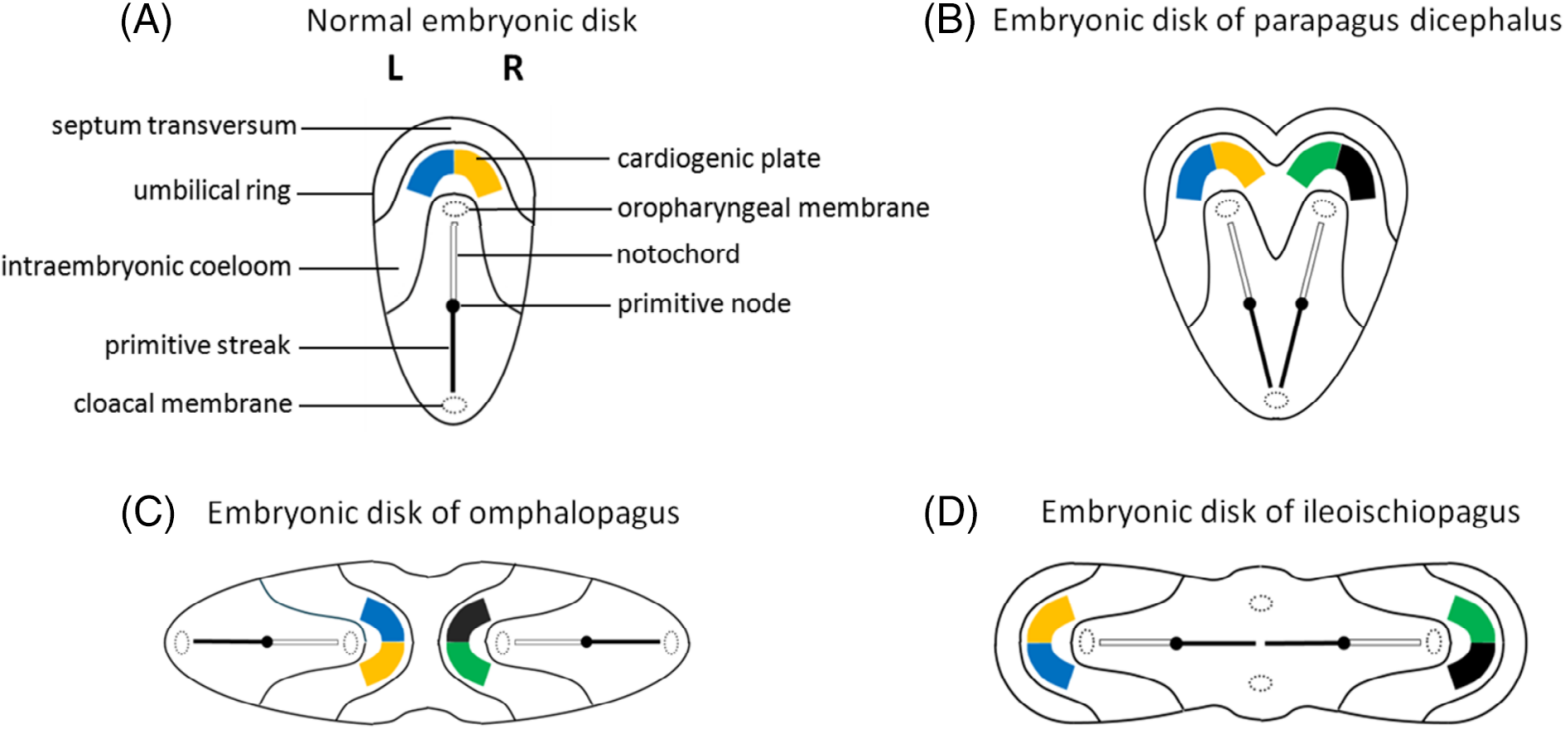
A, Schematic dorsal view of an embryonic disk at a late stage of gastrulation and normal configuration of early structures as depicted in many textbooks about embryology. B, When axial primordia (primitive streaks, nodes, and/or pits) are duplicated and located in a more or less parallel and angulated manner, with cranially located heart fields, laterally united twins will arise. The depicted embryonic disk shows the configuration of a parapagus dicephalus. Note the single cloacal membrane and the two oropharyngeal membranes. When this embryonic configuration persists, the ultimate phenotype will therefore include two heads, two hearts, two vertebral columns, and a single united lower body with a single umbilicus. C, When duplication of axially located primordia arise in an opposing manner and development proceeds, ventrally conjoined twins will arise. The depicted embryonic disk shows the possible configuration of an omphalopagus; only the diaphragm and liver are conjoined, resulting from the united septum transversum and umbilical ring. Note the presence of two oropharyngeal and cloacal membranes and two heart fields. The presence of two primitive streaks initiates duplicated notogenesis, ultimately leading to two complete vertebral columns and two complete neurulation processes. The ultimate phenotype of omphalopagi will include two heads, two hearts, and two lower bodies with a single umbilicus, as is clearly comparable with the depicted embryonic disk. D, Embryonic disk configuration of an ileoischiopagus. When two primordia arise in an opposing manner, although now with laterally located heart fields and oropharyngeal membranes and medially located cloacal membranes, caudal conjoined twins will arise. These twins have two separate hearts, two heads, two vertebral columns, and a conjoined and shared caudal area with a single umbilical cord. Adapted from Oostra RJ, Keulen N, Jansen T, van Rijn RR, Am J Med Genet, 2012, 80, 74–89. [Color figure can be viewed at http://wileyonlinelibrary.com]

Two embryological mechanisms—seen in ventrally, laterally, and caudally united twins—exist that both are responsible for adjustment and alteration of external and internal (embryological) morphology: neo‐axial orientation and interaction aplasia (Machin and Sperber, 1987; Spencer, 2003). Because of their exclusiveness in conjoined twins, these embryological adjustments could possibly imply certain etiopathogenetic clues about their origin. Whatever the true pathogenic mechanism of conjoined twinning may be, it is reasonable to assume that during early embryonic development, a certain “conjoined twinning event” occurs which results in aberrant hypoblast configurations that leads to duplication of the first visible initiations of gastrulation, being the primitive streak, node, and/or pit on a single cell mass or bilaminar embryonic disk. Hypothetically, the duplication and subsequent outgrowth of these morphogenetic primordia could occur in a direct opposite manner, resulting in ventrally and caudally conjunction types, or angulated and somewhat parallel configurations, resulting in laterally conjoined phenotypes (Fig. 3).

### Neo‐axial Orientation

**Figure 4:**
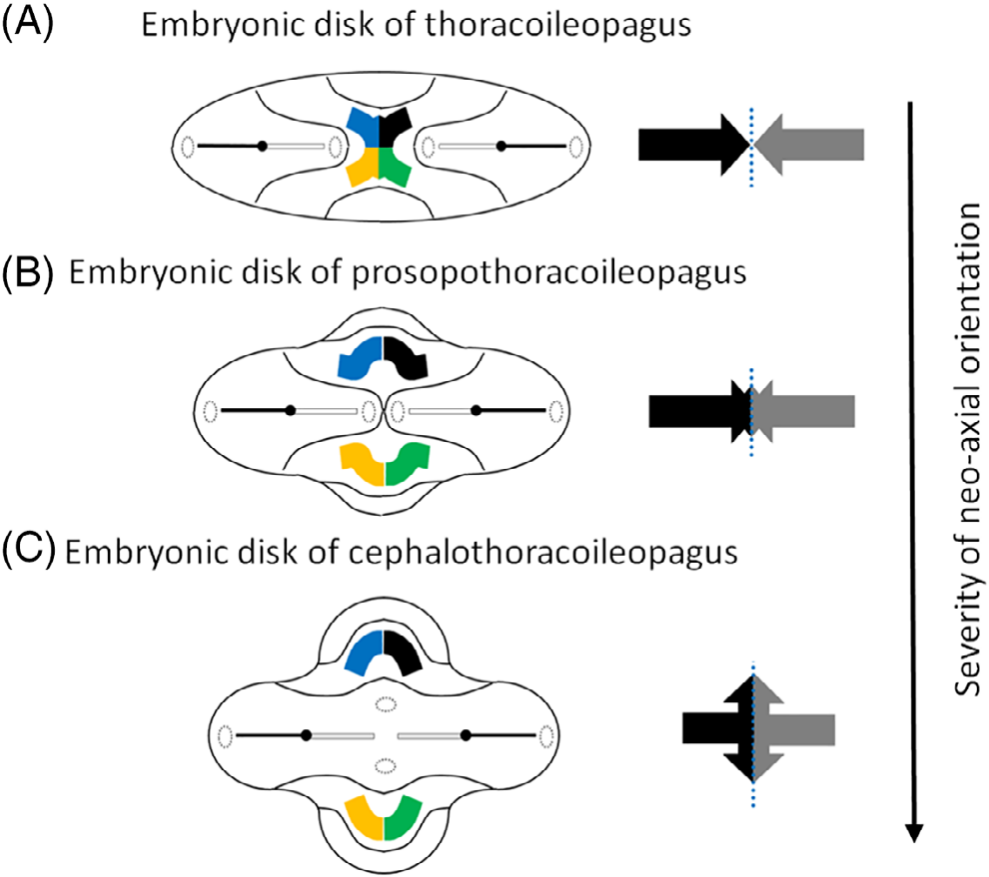
Embryonic disk models with opposing duplication of axial structures. A, Embryonic disk configuration of a thoracoileopagus with contiguous heart fields and neo‐axial orientation of structures derived from the anterior most parts of the embryonic disc, such as the sternums, livers, and diaphragms. This configuration will lead to a single complex and compound heart originated from cardiac primordia of both twins. Two separate heads and two lower bodies with a single umbilicus are found. B, Embryonic disk configuration of a prosopothoracoileopagus. When the initial reciprocal distance of two opposing primordia is more approximate than in thoracoileopagus, more intricate neo‐axial orientation will be initiated. This configuration will lead to neo‐axially oriented heart fields and thus to two compound hearts, in addition to the compound sternums, livers, and diaphragms. The presence of two separate oropharyngeal membranes, close to each other, will lead to two largely separate heads without neo‐axial orientation. C, Embryonic disk configuration of a cephalothoracoileopagus. If the initial distance between the opposing primordia is even more approximated, neo‐axial orientation will also involve facial and cranial structures. Note that the arrows represent the direction of relative growth of the embryonic disk. Adapted from Oostra RJ, Keulen N, Jansen T, van Rijn RR, Am J Med Genet, 2012, 80, 74–89. [Color figure can be viewed at http://wileyonlinelibrary.com]

Embryonic disks with two axial primordia in an opposing configuration—as is the case in ventrally and caudally conjoined twins—are subjected to neo‐axial orientations (Fig. 4). This embryonic adjustment refers to the mechanism by which opposing homologous structures are divided in the median plane after which the two halves will divert laterally. Compound organs and structures are thus formed by equal contributions of both embryos. The formed structures are located in a plane perpendicular to the original, thereby altering their original topographical location in a 90° axial rotation (Spencer, 2003). From a gross morphological point of view, two more or less normal structures are formed, although each half of these structures originally belongs to one of the twins. Neo‐axial orientation is demonstrated in all ventrally and caudally conjoined twins and is most dramatically demonstrated in cephalothoracoileopagi (joined at the head, thorax, and abdomen), in which two compound faces on opposite sides of a united head are seen (see Fig. 1E).

### Interaction Aplasia

**Figure 5:**
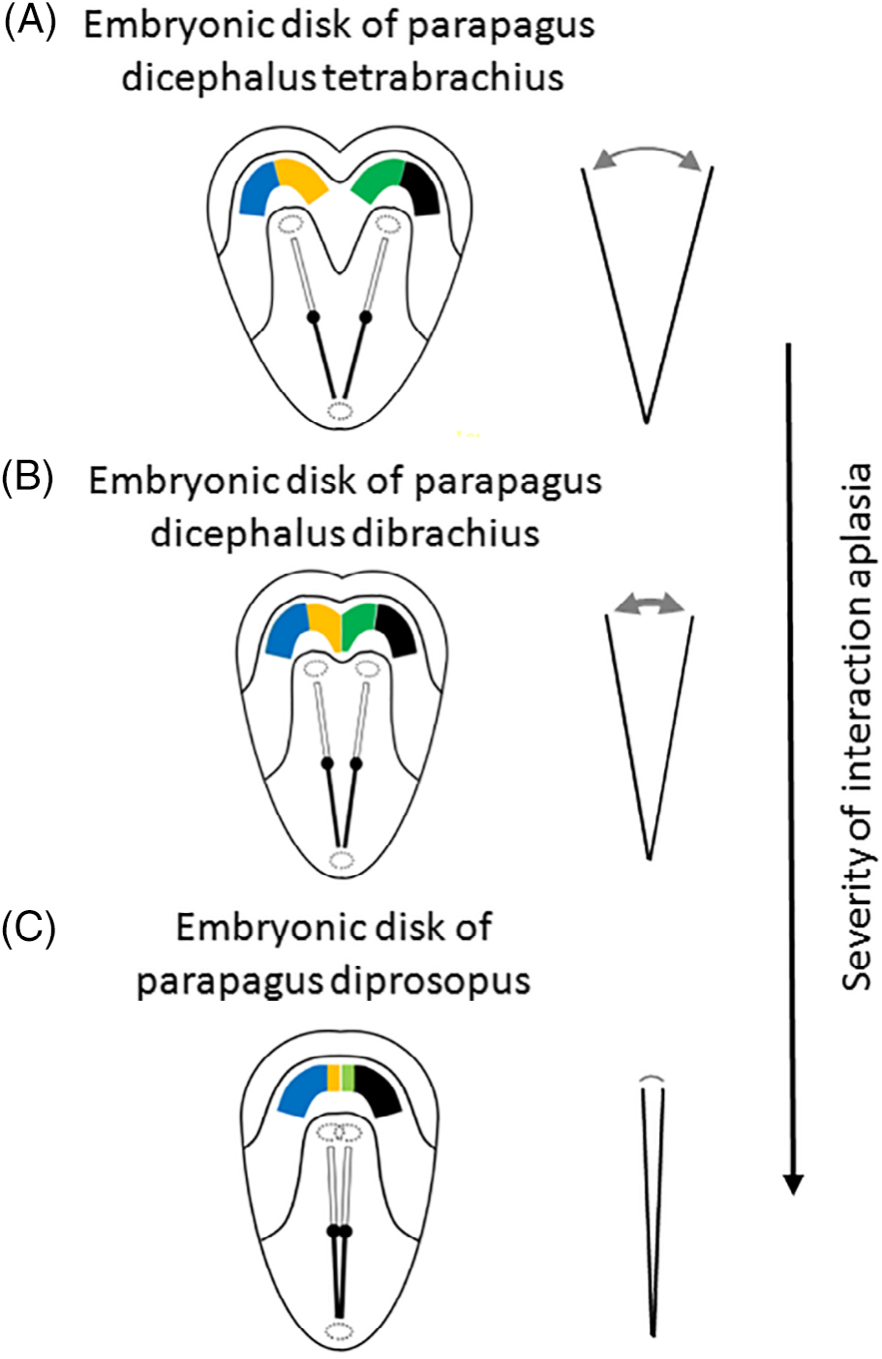
Embryonic disk models with two angulated axial structures. A, Embryonic disk configuration of a parapagus dicephalus tetrabrachius with two angulated axial primordia. Note the single cloacal membrane and the two, cranially located, heart fields and oropharyngeal membranes. Phenotypically, this configuration will lead to a parapagus twin with two heads, four arms, two separate hearts, more or less intricate junctions at the level of the lower thoraxes, diaphragms, and livers, and a single lower body with a single umbilicus. B, Embryonic disk configuration of a parapagus dicephalus dibrachius. If the angulation of the two axial primordia approximates more acute than in (A), their mutual distance is less and interaction aplasia will be more intense. Note that the heart fields of both twins become contiguous. This configuration will lead to a parapagus twin with two heads, two arms, a shared composite heart, profound junction at the level of the thorax(es), diaphragm(s), and liver(s), and a single lower body with a single umbilicus. C, Embryonic disk configuration of a parapagus diprosopus. If the initial position of the primordia is even more approximated, interaction aplasia of almost two complete body halves will occur. This configuration will lead to twins with a head with two (partial) laterally oriented faces on the ventral side and a more or less singular heart, diaphragm, and liver. Adapted from Oostra RJ, Keulen N, Jansen T, van Rijn RR, Am J Med Genet, 2012, 80, 74–89. [Color figure can be viewed at http://wileyonlinelibrary.com]

The second mechanical adjustment—again typical for nondorsally conjoined twins—is interaction aplasia. This mechanism is best demonstrated in laterally conjoined twins (i.e., parapagi). In contrast to the mechanism of neo‐axial orientation, occurring when primordia have opposite positions, interaction aplasia occurs when two primordia have any other mutual positions than exactly opposite, most typically when their positions are parallel. In interaction aplasia of contiguous primordia, organs and structures in the conjunction area fail to develop. The degree of aplasia depends on the approximation of the two primordia and their mutual angle. When approximation increases, interaction aplasia becomes more prominent (Fig. 5). Suppression of the structure and/or organ formation is assumed to result from aberrant concentrations of morphogens in and around the two longitudinal axes conflicting their concentration gradients and/or their (molecular) pathways (Levin et al., 1996). Primordia become obliterated by these overlapping gradients and subsequently fail to form a developmental field (Machin, 1993; Spencer, 2003).

## A PHENOTYPICAL CONTINUUM IN NONDORSALLY UNITED TWINS

**Figure 6:**
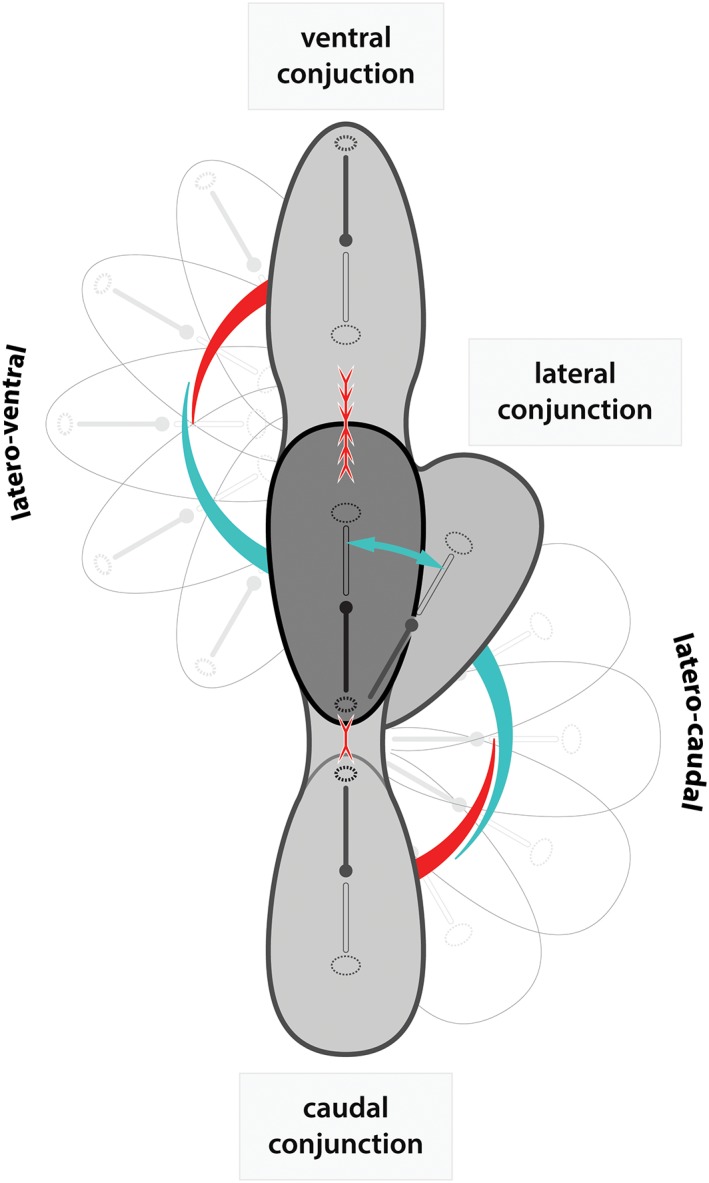
Schematic representation of a continuous model between lateral, ventral, and caudal united twins showing overlapping laterocaudal and lateroventral phenotypes. Interaction aplasia (indicated by the turquoise arrow) will decrease when the positions of the duplicated primordia become more opposite to each other. Interaction aplasia is thus absent in the caudal and ventral phenotypes. On the other hand, although neo‐axial orientation (red arrows facing each other) is absent in laterally united twins, this adjustment is profoundly present in the ventral and caudal conjunction group. However, the latter is affected much less severe. This is because embryonic growth is much greater toward the future head primordia than it is in caudal directions—as is indicated by the red arrows in the model. [Color figure can be viewed at http://wileyonlinelibrary.com]

**Figure 7:**
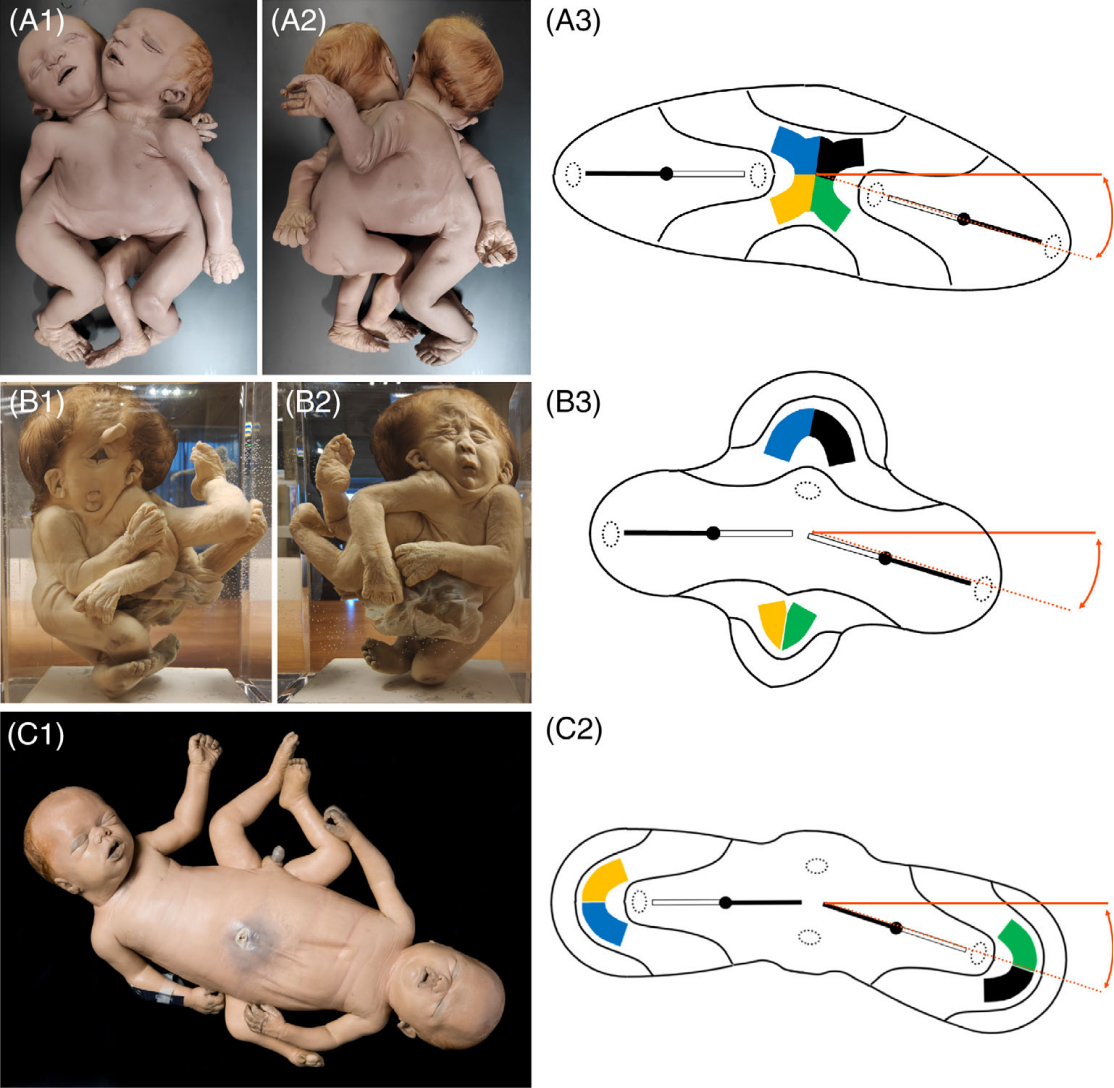
A, Thoracoileopagus tribrachius tetrapus twins with lateral deviations resulting in the formation of hypoplastic compound and composite structures. In this case, a composite arm forms at the concave side of the twins due to interaction aplasia of the compound shoulder girdle. B, Cephalothoracoileopagus twins with lateral deviations resulting in profound hypoplasia of craniofacial structures at the concave side of the twins, which is phenotypically reminiscent of holoprosencephaly. C, Ileoischiopagus tripus twins with lateral deviations resulting in a composite leg and hypoplastic penile structures at the concave side of the twins due to interaction aplasia of the compound pelvic girdle. All depicted specimens are from the Anatomical Museum in Nijmegen (The Netherlands). Figures of the embryonic disks are adapted from Oostra RJ, Keulen N, Jansen T, van Rijn RR, Am J Med Genet, 2012, 80, 74–89. [Color figure can be viewed at http://wileyonlinelibrary.com]

As stated above, the nondorsally united twins can all be included in a spectrum with infinite intermediate phenotypes and simultaneously concomitant neo‐axial orientation and interaction aplasia (Oostra et al., 1998). This is depicted in Figure 6. The intermediate phenotypes can all be extrapolated from the initial reciprocal distance and angle of the “duplicated axial primordia.” In that respect, it can be stated that no two pairs of conjoined twins are identical. For instance, many thoracoileopagi with axial primordia that are not exactly opposing each other show some degree of interaction aplasia, resulting in hypoplasia of compound organs and structures (e.g., junctions between two arms) on the concave aspect of the twins (Fig. 7A). Cephalothoracoileopagi with laterally deviating axes often show hypoplasia in one of the compound faces, mimicking the phenotype of holoprosencephaly (Fig. 7B). Finally, ileoischiopagi often show considerable caudolateral oriented variations and subsequent interaction aplasia, resulting in a composite lower limb and penoscrotal aplasia on one side of the conjoined pelvises (Fig. 7C).

## ETIOLOGY AND PATHOGENESIS OF CONJOINED TWINS

Many embryological theories are extrapolated by reasoning backward from late phenotypical stages to early embryological development (Opitz et al., 1979; Spranger et al., 1982). Although this method could be beneficial to exploit certain embryological explanations, it makes the enigmatic genesis of conjoined twins prone to conflux with regard to the actual cause and result. “You are not a twin because the inner cell mass splits, the inner cell mass splits because you are a twin” (Ronald, 2014). This quote reflects the exact problem in the everlasting enigma of imperfect twinning. What is the actual embryological cause? And what will be the final phenotypical result of this early defect? Therefore, the two paramount questions in respect to conjoined twins are: Why (etiologically) and how (pathogenetically) do these entities arise? Regarding the mechanism of conjoined twinning, there are currently two postulates: partial fission and secondary fusion. The fission theory, which assumes that conjoined twins originate around the primitive streak stage between days 15 and 17 of embryonic development (Sadler, 2010), is more profoundly postulated in textbooks, whereas the model of the secondary fusion is a widely accepted premise in the genesis of conjoined twins when exploring current research papers (Spencer, 2003).

### Fission Theory: Monozygotic Twinning and Incomplete Fission of a Single Embryo

The fission theory suggests that all types of monozygotic twins and conjoined twins are entities in a single etiopathogenetic continuum (Kaufman, 2004). Classically, depending on the time of fission, separate monozygotic twins can be divided into three entities (Czyz et al., 2012; McNamara et al., 2016). The developmental stage at which splitting occurs would determine chorionicity and amnionicity (Herranz, 2015). In the morula stage, splitting is thought to result in two genetically identical blastocysts. Each blastocyst will implant separately, ultimately leading to a dichorionic‐diamniotic (DC‐DA) placentation. This type of monozygotic twinning accounts for approximately 18%–36% of all monozygotic twins and is thought to occur within 3 days of embryonic development (Singh et al., 2002). If fission of the embryoblast (without interfering the trophoblast) would occur in the peri‐implantation period—after the third but before the seventh day of development—two embryoblasts in a single blastocyst would form and hence a monochorionic‐diamniotic (MC‐DA) placentation. This type accounts for about 60%–80% of monozygotic twins (Singh et al., 2002). The third developmental stage at which fission could occur is the most uncommonly encountered group of monozygotic twins: this type is seen in about 1% to 4% of human monozygotic twins (Kaufman, 2004). Splitting is thought to occur shortly before or during the formation of the primitive streak (around Day 15 of development). The bilaminar embryonic disk is believed to “split in two,” giving rise to two embryos which develop within a single amniotic cavity and inherently show a monochorionic‐monoamniotic (MC‐MA) placentation. When fission occurs after Day 15 of development—when there is presence of a bilaminar embryonic disk as well as three extra embryonic spaces (amniotic cavity, primitive yolk sac, and chorionic cavity) and a single (caudally located) connecting stalk—it is assumed that fission will be incomplete and will subsequently give rise to the various forms of conjoined twins (Kaufman, 2004). Noteworthy is that the postzygotic fission model places all conjoined twins in a single receptacle (together with all three types of separate monozygotic twinning), and extensive embryonic development is only minimally taken into consideration.

Several mechanisms have been proposed that could explain the occurrence of splitting as the cause of separate monozygotic twinning. Blickstein and Keith (2007) proposed that a small proportion of oocytes might have an inborn tendency to undergo splitting upon fertilization, leading to the constant prevalence of spontaneous monozygotic conceptions among different populations. The revolutionary idea of an imprinted twinning gene needs further investigations (Shur, 2009). But indeed, monozygotic twinning occurs at a relatively constant rate of three to five in 1,000 births worldwide, supporting the view that it represents a random and/or genetic event (Tarlatzis et al., 2002). A paper by Liu et al. (2018) described a four‐generation pedigree of monozygotic female twins revealing novel genetic variants specific to monozygotic twins in the X chromosome.

Apparently, there are certain factors (e.g., environmental, mechanical, and genetic) that potentially play a key role in the occurrence of different types of monozygotic twins. Proposed triggers for splitting include gene mutations, abnormalities in cell surface proteins, and abnormalities in the formation of the zona pellucida (Kilby et al., 2006; Mercan et al., 2011; Jim and Berkovich, 2015). Increased frequency of monozygotic twins is observed after infertility therapy such as intracytoplasmic sperm injection (Sills et al., 2000; Song et al., 2017; Hviid et al., 2018), indicating a possible association of zona pellucida damage and monozygotic twinning. Enders (2002) described that one possible cause for the increase in monozygotic twinning following in vitro fertilization such as assisted hatching is the constriction of the inner cell mass during mechanical hatching of the blastocyst from the zona pellucida as opposed to the natural digesting that occurs in vivo. Indeed, in vivo developed mouse cleavage stages following focal damage to the zona pellucida frequently yielded two blastocyst‐like structures on subsequent recovery from the uterus (Malter and Cohen, 1989). This phenomenon can be explained by partial herniation of the blastocyst followed by its shearing from the zona pellucida. These herniated trophectodermal vesicles may include inner cell mass tissue and subsequently can form an additional blastocyst (Malter and Cohen, 1989).

### Fusion Theory: Secondary Fusion of Two, Initially Separate, Embryonic Disks

**Figure 8:**
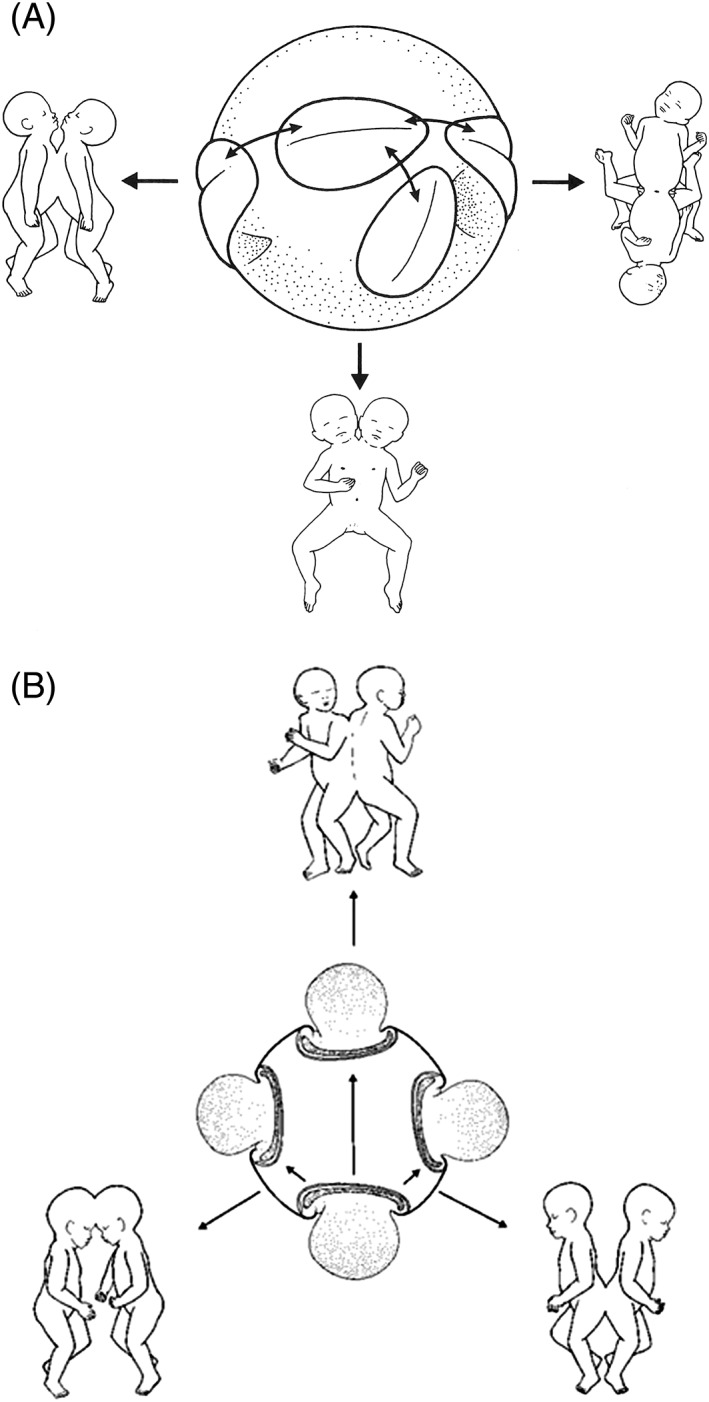
The spherical theory as the etiological basis for conjoined twins devised by Spencer. A, When two embryonic disks lie adjacent to each other and “float” on the outer surface of a spherical yolk sac, ventral, lateral, or caudal conjunction types could occur. B, When two embryonic disks “float” on a shared amniotic cavity, the possible secondary fusion of two, initially separate, primitive neural folds can occur, resulting in dorsally united twins. Note the presence of two yolk sacs. Reused with permission from Spencer R, Clin Anat, 2000, 3, 36–53, John Wiley and Sons.

In contrast to the fission theory, the fusion theory—predominantly embraced in current research papers—suggests that conjoined twins result from two, initially separate monozygotic embryos, which coalesce and become secondarily and homologously fused (Guttmacher and Nichols, 1967). This fusion theory was espoused by Spencer (2003) and is now a widely accepted theory, cited in virtually every paper on this topic. Spencer (2003) proposed that conjoined twins originate when the inner cell mass divides (implying an early fission) during the first week after fertilization into two separate monozygotic embryonic primordia staying close enough together to share either the amniotic cavity or the yolk sac. When these embryos continue their rapid growth, they might come in contact with one another and become reunited to result either in ventrally, laterally, caudally, or dorsally conjoined twins. Spencer (2000) substantiated the concept of secondary fusion with a theoretical model called the “spherical theory.” This model delineates that two monozygotic embryonic disks lie adjacent to each other and “float” on the outer surface of a spherical yolk sac resulting in an—always homologous—ventral, lateral, or caudal configuration. When two monozygotic embryonic disks “float” on a shared amniotic cavity, the possible secondary fusion of two, initially separate, primitive neural folds can occur, resulting in dorsally united twins (Fig. 8). In addition, Spencer (1992) described that secondary fusion will not occur randomly and that intact skin will not fuse with intact skin. Union only occurs where surface ectoderm is either absent (primordia of the heart and septum transversum) or is destined (preprogrammed) to undergo apoptosis (neural tube, the oropharyngeal and cloacal membranes) or is influenced by differential growth (the periphery of the embryonic disk). Furthermore, union always occurs in the midline, “the two lateral halves of specific structures of one embryo united to the opposing halves of the same structures of the other embryo” (Spencer, 2003). According to Spencer (2003), the presence of supernumerary umbilical vessels and the presence of two umbilical cords in dorsally conjoined twins is a strong argument for the fusion theory of two initially separate embryonic disks which coalesce and will fuse secondarily.

## “CROWDING” THEORY: INDUCTION OF TWO AXIAL PRIMORDIA

In addition to the fission and fusion theories, a third conjecture to explain conjoined twins may be the initial “crowding and thereby duplication of morphogenetic potent primordia” (Kapur et al., 1994; Bidondo et al., 2016; Martyn et al., 2018). Interestingly, already in 1866, Fisher described duplication of the primitive streak in one embryonic cell mass as the cause of conjoined twinning (Fisher, 1866). Wilder (1908) concluded that there is “neither a fusion of parts already formed nor a gradual development from the normal towards the abnormal during embryonic life, but the parts appear double or reduced from their first appearance, and their development is controlled in the same way as are the bilateral structures and other architectural characteristics of normal beings.” Moreover, Ysander and Wikstrom (1925) concluded that “the manner in which twins will be definitely joined depends on the distance between the two developing centers, their independence of each other, and the angle between their polar axes.” The “crowding of organizers” presumes the early induction of two instead of one multifaceted organizer on the surface of one embryonic cell mass. When applied to nondorsally united twins, the initial reciprocal distance and mutual position determines the site and degree of conjunction. Depending on the configuration of the initial “duplications” (opposite or angulated), the neo‐axial orientation and/or interaction aplasia occurs (Machin, 1993; Spencer, 2003; Oostra et al., 2012). The premise of initial duplication of certain “axial primordia” is strengthened by many experimental studies. Since the initial transplantation experiments by Spemann and Mangold (1924), amphibians, and especially *Xenopus*, have served as model systems for the analysis and manipulation of axis formation in the vertebrate embryo (Tisler et al., 2017). In subsequent decades, experimentally induced duplication of organizing centers in mammals and nonmammals have been described abundantly (Table 1). In addition, Ziv et al. (1992) showed that mesoderm induction is mediated through morphogens distributed in a gradient manner and suggested that during normal development, only one axis is obtained because of carefully controlled inhibitory processes. Seleiro et al. (1996) described genes of the transforming growth factor β superfamily as the earliest steps of developmental patterning in vertebrates, and Beddington (1994) suggested that the node can serve as a “stem cell” source of axial mesoderm. Besides describing axes duplication, Pöpperl et al. (1997) stated that a number of secreted protein factors—such as certain Wnt family members, noggin and Vg1—can induce the formation of a second axis in *Xenopus*. The embryos exhibited two different forms of axis duplication: either the axes were in opposing orientation, giving a head‐to‐head duplication or they were angulated and fused caudally. These configurations are exactly the same as observed in human conjoined twins. Moreover, it has been shown that *axin*—encoding for an inhibitor of the WNT‐signaling pathway—regulates embryonic axis formation in mouse and *Xenopus*. Besides induction of axial duplications, Perea‐Gomez et al. (2002) demonstrated that *Nodal* antagonists in the AVE prevent the formation of multiple primitive streaks. Nodal is known to play an important signaling role from the node, in the anterior primitive streak to the lateral plate mesoderm (Kawasumi et al., 2011). In addition, Gardner (2001) demonstrated that the axis of polarity of the developing mouse embryo may be established as early as in the zygote or during the first cleavage stage. McCrea et al. (1993) described that embryonic axis formation initiates before major activation of the zygotic genome. Noteworthy is that before zygotic genome activation, early mammalian development relies on maternal effect genes to orchestrate the oocyte‐to‐embryo transition (Lu et al., 2017). It can be even hypothesized that these critical mRNAs could be dislocated, inducing polarity changes and subsequent duplications (Driever and Nüsslein‐Volhard, 1988; Goldman and Gonsalvez, 2017). Vandenberg and Levin (2012) found that apical–basal and planar polarity proteins are required for left–right axis orientation in *Xenopus*. Studies in Table 1 clearly indicate that it is possible to experimentally initiate duplicated axial structures such as primitive streaks, nodes, or notochords on one embryonic disk and obtain phenotypes that are indistinguishable from conjoined twinning.

**Table 1:** Overview of Recent Studies (Since 1991) in Which Duplicated Axial Structures Are Found After Molecular and Genetic Alterations

Author and year of publication	Species	Short description of their findings
Sokol et al. (1991)	*Xenopus*	Injection of Wnt mRNA induced complete axis duplication
Ziv et al. (1992)	Chick	Produced ectopic axes after injecting Activin containing medium
Beddington (1994)	Mouse	Induced ectopic notochords by implanting grafts of transgenically marked mid‐gastrulation nodes
Karnovsky and Klymkowsky (1995)	*Xenopus*	Injection of RNA encoding an epitope‐tagged form of plakoglobin induced axis duplication
Toyama et al. (1995)	Zebrafish	Injection of *Nodal* mRNA produced duplication of the notochord and somites
Seleiro et al. (1996)	Chick	Induced a complete second axis after implanting grafts with Vg1 protein
Molenaar et al. (1996)	*Xenopus*	Injection of β‐catenin RNA consistently induced axis duplication
Pöpperl et al. (1997)	Mouse	Ectopic expression of Cwnt8C caused axis duplication in *b‐actin‐Cwnt8C* transgenic mice
Zeng et al. (1997)	*Xenopus*	Suppression of wild‐type *Axin* resulted in duplication of the body axis
Nascone and Mercola (1997)	*Xenopus*	Microinjection of mRNAs encoding Wnt signaling pathway components *wnt8* or β‐catenin duplicated the inductive properties of the Niewkoop and Spemann regions and created conjoined twins
Fang et al. (2000)	Frog	Ectopic injection of noggin RNA in blastomeres induced complete duplications of axes including heads and eyes
Perea‐Gomez et al. (2002)	Mouse	Demonstrated that *Cerl* ^*−**/**−*^ *;Lefty1* ^*−**/**−*^ compound mutants developed ectopically primitive streaks
Merrill et al. (2004)	Mouse	Demonstrated that in *Tcf3* ^*−**/**−*^ mutants duplication of nodes and notochords occurred
Tisler et al. (2017)	*Xenopus*	Induced conjoined tadpoles after injection of Wnt‐pathway components into the ventral marginal zone of cleavage stage embryos

## DISCUSSION

Normal human pregnancy concerns single offspring; it is therefore that (imperfect) twinning is in itself a congenital anomaly (Weber and Sebire, 2010). Although—relatively many—gross congenital anomalies have a known cause, the etiology and pathogenesis of (conjoined) twinning remains enigmatic. Many case reports exist on the topic of conjoined twinning, especially regarding separation and preoperative and postoperative management. Interesting is the underrepresentation of papers which (extensively) discuss their potential etiology and pathogenesis or correlate human embryology in its delineated contemplations. Apart from the “crowding” concept presented in this article, there are currently two conjectures for their possible genesis: partial fission versus secondary fusion. These mutually exclusive hypotheses are—curiously enough—widely spread in medical textbooks on embryology and cited abundantly in current literature. Despite their purported plausibility, they show clear omissions, lack a substantiated (theoretical) correlation with (human) embryological development, and are not unequivocally demonstrated experimentally. Furthermore, traditional assumptions such as all conjoined twins having a common etiology and pathogenesis are often adopted without any critical appraisal. This could cause erroneous theories to evolve into accepted and apparent factual etiological and pathogenetic models. These models should be critically reviewed and reevaluated to break the current paradigmatic stalemate.

### Comments on the Fission Theory

The fission mechanism behind monozygotic twinning is still poorly understood. Corner (1955) stated that selective cellular death can act as a dissecting knife dividing the embryo into two. A paper by Herranz (2015) argued that the commonly accepted Corner model (Corner, 1955) of postzygotic fission lacked scientific proof. He stated that factors initiating cleavage are unspecified, coexistence of separate embryos within a single zona pellucida seems unlikely, postzygotic splitting becomes more unlikely with the passage of time, and splitting has never been observed in vitro. However, a paper by Kyono (2013) found evidence from in vitro fertilization studies that monozygotic DC‐DA twins would occur at the blastocyst stages and not during early morula stages, doubting the long held credo that DC‐DA twins would develop after embryo splitting in the early stages of embryonic development. Furthermore, Herranz (2015) proposed a new theory to explain the timing of monozygotic twinning. Monozygotic twinning would be a fertilization event; “due to an alteration of the zygote–blastomere transition, the first zygotic division, instead of producing two blastomeres, generates twin zygotes. Second, monochorionicity and monoamnionicity would not depend on embryo splitting, but on fusion of membranes.” Critical notes on the paper of Herranz were espoused by Denker (2015) and Gardner (2014), which both concluded that the traditional Corner model and Herranz model were unsubstantiated. However, none of the above authors postulate an alternative explanation.

**Figure 9:**
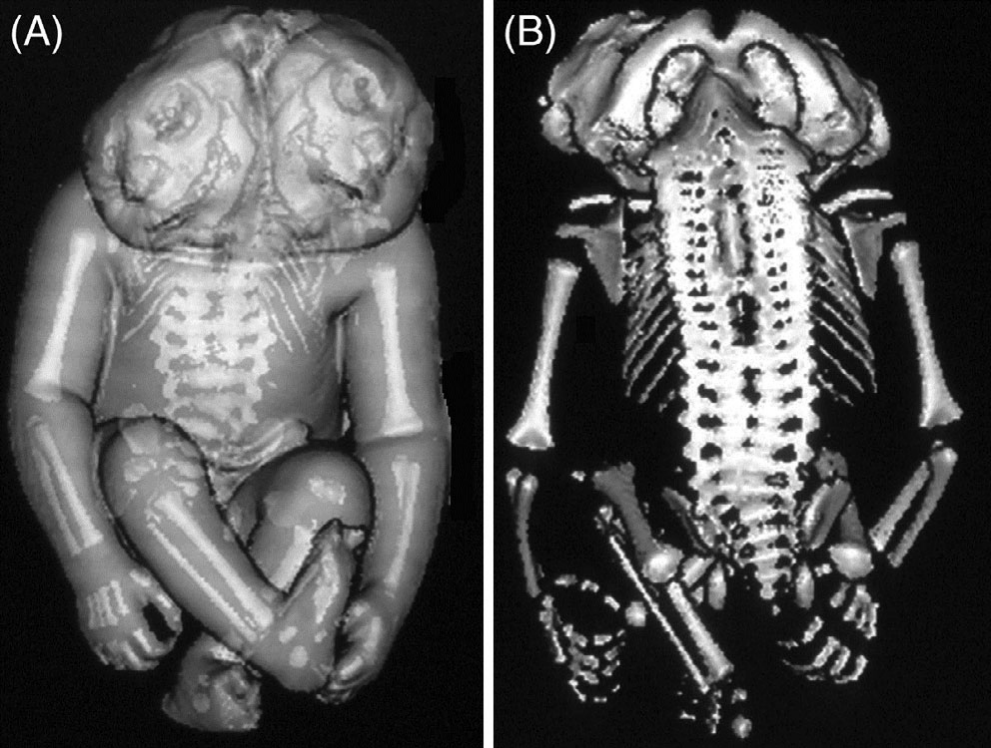
Digitally reconstructed images of a spiral computed tomography from a parapagus diprosopus from the Vrolik collection in Amsterdam (The Netherlands). A, Ventral view of the specimen with the outer contour combined with the reconstructed skeleton. B, Dorsal view showing complete duplication of the vertebral column. Note the concomitant craniorachischisis which is often present in parapagi diprosopi.

With respect to the different types of monozygotic twins, the veracity of the currently used fission model remains rather debatable. However, this ubiquitous model could indeed be plausible in DC‐DA and MC‐DA twins. This argument can be strengthened by the fact that after, for example, assisted fertility treatment—which often yields multiple gestations—both monozygotic DC‐DA and MC‐DA twins occur (Wehbe et al., 2003; Yanaihara et al., 2017). However—and to the best of our knowledge—we did not come across any reports of MC‐MA twins after infertility therapy, indicating that the model of postzygotic fission is perhaps not applicable for MC‐MA twins. The only cases reported like MC‐MA are those resulting from a dividing membrane in an MC‐DA gestation which ruptures, creating a functional MC‐MA configuration and a “pseudomonoamniotic” gestation (Patil et al., 2015). A paper by Galjaard et al. (2014) reported on two intermediate forms of chorionicity and amnionicity that may arose due to zygotic cleavage within the time interval just between dichorionic and monochorionic and diamniotic and monoamniotic twinning. Although the presence of pseudo and intermediate types of MC‐MA twins (which are truly exceptional cases), we feel inclined to suggest that zygotic fission could be applicable in DC‐DA and MA‐DA twins but is not necessarily etiologically responsible for the formation of MC‐MA twins and conjoined twins, and that the spectrum between these entities is not immediately obvious. It is noteworthy that the acclaimed homology in the etiopathogenesis of monozygotic and conjoined twins is currently only based on the congruent configuration of its embryonic membranes (McNamara et al., 2016). Although this premise is true, it is not because of this peculiarity that they necessarily have a common etiological background (see further). The model of (in)complete fission is rather hard to imagine embryologically, and some questions remain inconclusive. For instance, it remains a mystery why fission occurs at these specific days of development? Is fission a time‐specific event or could this process cease after a certain threshold of development? What causes partial zygote splitting after Day 15 of development and is it even possible at this stage? And how can the amniotic epithelium insinuate between (partially) fissioned hypoblasts and epiblasts? In addition to the fission model being plausible in DC‐DA and MC‐DA twins, the assumption that incomplete fission could explain conjoined twinning is merely based on the Y‐shaped contour of laterally conjoined twins, with their “split” and duplicated upper body halves and singular lower body half, creating the illusion of incomplete fission confined to the anterior part of the embryonic disk as its pathogenesis. It truly concerns that an illusion is underpinned by the fact that the—on external examination—seemingly singular and hence “unsplit” lower body half of even the most intricately conjoined parapagus twins, being diprosopus, in fact shows longitudinal duplications down to the caudalmost end of the vertebral column (Fig. 9). Moreover, the explanatory potential of this illusion fails when applied to ventrally and caudally conjoined twins. Because, as we have shown throughout this article, all nondorsally conjoined twins are part of a phenotypical continuum and are subsequently affected by the same embryological adjustments (neo‐axial orientation and/or interaction aplasia), this means that incomplete fission cannot be the cause of laterally nor of any other type of nondorsally conjoined twins.

### Comments on the Fusion Theory

Although we did not come across any literature concerning experimental in vitro fusion of developing placental mammals, studies generating parabiotic zebrafish embryos indicate that it is physically possible to fuse blastula stage zebrafish embryos ultimately creating parabiosis which develop as partially fused embryos sharing a common blood circulation (Demy et al., 2013). Noteworthy is that fusion always required (micro)surgical procedures inducing an artificial component (Kamran et al., 2013). A study by Gianasi et al. (2018) detected for the first time that a sea cucumber (*Cucumaria frondosa*) could undergo natural zygote fusion in hatched blastulae.

In addition, according to Spencer (2003), the presence of supernumerary umbilical vessels and the presence of two umbilical cords in dorsally united twins are an important argument for the fusion theory of two initial separate embryonic disks. However, only a small percentage of nondorsally conjoined twins show supernumerary vessels. Many twins show normal configurations of the umbilical vessels or even less than three vessels (Konstantinova, 1976). The connecting stalk will be formed on Days 13–14 by condensed extraembryonic mesoderm in which subsequently umbilical vessels will develop and into which the allantois grows (Müller, 1848). Strangely, Spencer (2003) stated that conjoined twinning originates in the first week after fertilization. According to the fission theory of monozygotic twinning—described above—if complete splitting occurs in the first week of development, a monochorionic‐diamniotic placentation will occur. With this configuration, it is impossible to get secondarily fused twins as the amniotic membranes would interfere within this process. Furthermore, in this period, gastrulating processes have not yet occurred so there is neither a bilaminar or trilaminar embryonic disk nor a connecting stalk with umbilical vessels. The initial presence of two caudally located body stalks is inherent if the secondary fusion takes place after or around 14 days of embryological development. Many reports describe that conjoined twins arise after Day 14 of development, independent if fusion or fission might have occurred. It is however difficult to envision how the process of two separate embryonic disks, with two connecting stalks and all their embryological primordia, unite to form one seemingly “fused” individual with one umbilicus and often profound embryological adjustments. If this process would actually happen around the primitive streak stage of development, one would expect duplication of umbilical vessels and paraumbilical structures in all conjoined twins.

Although we excluded nonsymmetrical twins, a paper by Logroño et al. (1997) has to be noted. They found three different alleles in four loci at the site of junction of a parasitic conjoined twin with fluorescent in situ hybridization techniques. It is noteworthy because it was this finding which was the most decisive argument for secondary fusion (Spencer, 2003). Although these findings could be correct, dizygosity is not immediately implicated when finding genetic differences between members of (parasitic) conjoined twins nor does it imply that fusion is the cause of their conjunction. Traditionally, it is presumed that monozygotic twins are genetically identical and subsequent phenotypical discordances are ascribed to environmental influences alone (shared or non‐shared), thereby altering and modifying the expression of the otherwise identical genetic endowment (Gringras and Chen, 2001). Recent insights indicate that this explanation is far too simple (Czyz et al., 2012). Genetic divergence due to post‐zygotic point mutations does occur (Acuna‐Hidalgo et al., 2015). Gringras and Chen (2001) reviewed genetic alterations in monozygotic twins and found heterokaryotypical divergence, chromosomal mosaicisms, epigenetic modifications such as DNA methylation, histone acetylation, and skewed or nonrandom X‐inactivation causing discordance in monozygotic twins. Moreover, divergent epigenetic modifications can lead to differential expression of inherited disease genes (Petronis et al., 2003; Castillo‐Fernandez et al., 2014). Furthermore, phenotypic discordance in monozygotic twins may, in part, be caused by de novo mutations of copy‐number variants and copy‐number variants mosaicisms (Bruder et al., 2008). Copy‐number variants account for a major portion of the genome and are strongly polymorphic and relatively unstable, with mutation rates 100 to 10,000 times higher than those for single base substitutions (Itsara et al., 2010). Additionally, unequal exchange of cells during gestation might potentially lead to discordant fetomaternal microchimerism and thus possibly induce discordances in monozygotic twins (Gringras and Chen, 2001). Evidence has been accumulating showing that spontaneous chimerism is far more common than previously realized (Boklage, 2009). It is therefore not surprising that monozygotic twins can show a high degree of discordance for complex genetic traits and disorders. Taking the above mentioned in mind, it is imaginable that genetic differences occur in members of conjoined twins, irrespective of their pathogenesis; because of its monozygotic nature, subtle gene differences do not directly imply dizygosity (Shur, 2009).

The theory of secondary fusion and its accompanying spherical etiology, as postulated by Spencer (2000), is rather difficult to (embryologically) envision in the nondorsally united twins, giving this model less credibility. Moreover, it is intriguing to question—in accordance with the spherical theory—how conjoined twins can be affected so dramatically by neo‐axial orientation and/or interaction aplasia when two (complete?) embryological entities coalesce. Secondly, it remains a mystery why some zygotes float on a shared yolk sac and have two amniotic cavities and others float on a shared amniotic sac with two yolk sacs (Spencer, 2003). No rational (embryological) explanation is present to validate this assumption. Finally, it is unclear how and why initially separated embryonic disks would coalesce homologously.

Interestingly Spencer stated, with respect to laterally conjoined twins, that “the two lateral halves of specific structures of one embryo united to the opposing halves of the same structures of the other embryo” (Spencer, 2003), but strangely enough she subsequently admits that the secondary fusion theory is rather hard to imagine within the lateral group and cannot be immediately explained within this model. Because all nondorsal types of conjoined twinning form a phenotypic continuum, as we have demonstrated above, precluding one type from this explanatory postulate makes it highly unlikely that it remains applicable to the other types. As with the fission theory, the concept of secondary fusion as the causative explanation for nondorsally conjoined twins is based on an illusion, in this case created by superficially connected ventrally and caudally conjoined twins. However, the illusion fails as soon as one is confronted with more intricate neo‐axial orientation and especially with interaction aplasia.

However, the secondary fusion theory could be the underlying mechanism in dorsally conjoined twins which show several characteristics, unique to this group, which may suggest an etiopathogenesis that fundamentally differs from that of other types of conjoined twinning. First, dorsally conjoined twins always have two separate umbilical cords and show no phenotypic overlap with other conjoined twinning groups. Secondly, there is no or very restricted neo‐axial orientation and/or interaction aplasia, in that a low percentage show superficially shared brains and dural venous sinuses (Stone and Goodrich, 2006). In addition, dorsally united twins frequently show a nonhomologous union such as a temporoparietal or occipitofrontal union in craniopagus (Walker and Browd, 2004). This is in contrast to the ventrally, laterally, and caudally conjoined twins, which are always united in a homologous fashion. As proposed by Spencer (2003), dorsally conjoined twins could theoretically arise when two—initially separated—rapidly growing monozygotic MC‐MA embryos get in mutual contact with the still open parts of their neural grooves and become secondarily fused. Assuming that secondary fusion is the causal mechanism for dorsally conjoined twinning, three different entities can be discerned which depends on the time and specific site of fusion. Fusion at the cranial neuropore forms craniopagi, fusion at the caudal neuropore results in pygopagi, and fusion in the midportion of the neural tube will create rachipagi.

The presence of two separate umbilical cords implies the presence of two primordial connecting stalks on Day 14 of embryological development and thus two initially separate embryonic disks. Furthermore, the clear absence of pronounced morphological adjustments in the plain of conjunction and the occasional nonhomologous conjunction all plead for secondary fusion at the sites of neural tube closure of two initially separate embryos. Spencer (2003), assuming a single pathogenetic mechanism for all types of conjoined twinning, concluded that “The dorsally united twins present the most compelling argument for the fusion theory—and against fission—as the origin of conjoined twins” (Spencer, 2003), a view point that rapidly gained ground.

**Figure 10:**
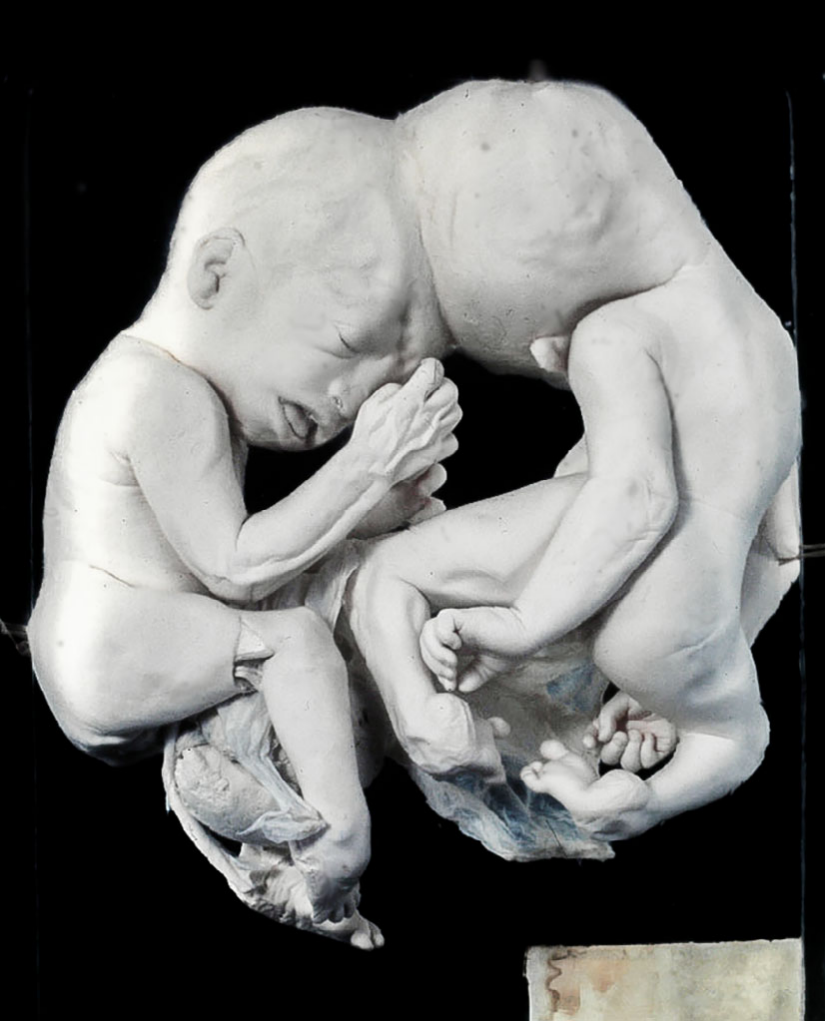
Cranioamniopagus twin from the Vrolik collection in Amsterdam (The Netherlands). [Color figure can be viewed at http://wileyonlinelibrary.com]

Noteworthy is one truly exceptional case of a craniopagus, described by Bolk (1926) (reviewed by Oostra et al. (1998)), historically diagnosed as cranioamniopagus. This twin was united in two separate sites: a nonhomologous union at the head was accompanied by an overarching omphalocele with concordant cloacal exstrophy (Fig. 10). In this case, a secondary fusion of the cranial neuropore could have occurred in an initially very superficially conjoined xipho‐omphalopagus; this site of union may have acted as a “hinge point” creating exactly the right distance and mutual opposability between the two twins to facilitate the occurrence of secondary fusion at the site of the cranial neuropores. These hinge points are inherently absent in more profoundly united ventrally as well as in laterally or caudally conjoined twins.

### Initial Axial Duplications May Be Responsible in the Genesis of Ventrally, Laterally, and Caudally Conjoined Twins

With the rejection of both the fusion and the fission theories as causative explanations, we propose that initial duplication of axially located morphogenetic potent primordia in one inner cell mass is the initiating factor in the genesis of nondorsally conjoined twins. Moreover, we also propose this mechanism to be responsible for (at least some cases of) separate MC‐MA twinning, in which we assume the initial reciprocal distance between the axial primordia to be large enough to prevent mutual developmental interference from occurring. Conjoined and separate MC‐MA twinning are equally rare with prevalences of 1%–4% of all monozygotic twins (Kaufman, 2004). But more compelling is the rare but repeatedly reported occurrence of MC‐MA twins with a single placentally inserted but bifurcated umbilical cord, connected with two separate MC‐MA twins (reviewed by Fraser et al., 1997), which could be interpreted as a transitional twinning type between separate and conjoined MC‐MA twins. Because we demonstrated in the previous paragraphs the plausibility of DC‐DA and MC‐DA monozygotic twinning to result from fission of the early embryoblast but excluded this mechanism as causative for any form of conjoined twinning, the pathogenic connection made here between MC‐MA monozygous twins and nondorsally conjoined twins implies that monozygous twinning is a heterogeneous phenomenon.

Interestingly, this “molecular and morphological crowding” of axial primordia has been abundantly described in animal experimental studies as ectopic or duplicated axial structures in a single entity, including the primitive streak, node, or notochord (see references in Table 1). These experiments induced duplications by altering various secretion and transcription factors all involved in embryonic axis formation. Curiously, these findings have rarely been correlated with the genesis of human conjoined twins. It can be assumed that duplicated primordia are localized in a certain (pre)destined pattern, both following their own fate while inducing their own signaling pathways (Tabata and Takei, 2004). Subsequently, these signaling pathways could potentially interfere with each other and create dysmorphological phenotypes (Levin et al., 1996; Gilbert‐Barness et al., 2003). It is known that the loss of functional mutations of genes expressed by the AVE results in the formation of extra primitive streaks (Schoenwolf et al., 2009).

## CONCLUSION

A pitfall in the ongoing etiopathogenetic debate on the genesis of conjoined twins is the fact that different types of conjoined twins are classically placed in one overarching receptacle. This approach has seriously hindered the quest for explanatory models.

We have shown that all nondorsally conjoined twins are part of a single phenotypical spectrum and probably have a single etiology and pathogenesis and that both the fission as well as the secondary fusion hypotheses to explain the pathogenesis of nondorsally conjoined twins are based on illusions.

Although the following needs further empirical evidence, we consider that the etiopathogenesis of dorsally united twins could be attributed to secondary fusion of two initially separate monozygotic twins. Based on what is presented in this article, we propose that initial duplication of axially located morphogenetic potent primordia could be the initiating factor in the genesis of ventrally, laterally, and caudally conjoined twins as well as monozygotic MC‐MA twins. The model of postzygotic fission could be possible in the genesis in DC‐DA and MC‐DA twins. However, one must be aware, although we state that MC‐MA twins and conjoined are part of a continuum, that these entities could still be etiologically heterogeneous. In addition, it is conceivable that very little additional diagnostics are performed after the birth of conjoined twins—perhaps because it is obvious that it concerns a united twin with a supposedly perspicuous etiology when one looks perfunctory to literature. However, new cases should be critically evaluated with addition radiological imaging and genetic diagnostics (Kompanje, 2006). Finally, determination of the chorionicity and amnionicity in new cases is crucial to proof our propositions.

## LIMITATIONS OF THIS STUDY

The first footnote in this study is the lack of prior comprehensive appraisals on the correlation of human conjoined twins and an elaborate view on early embryogenesis. In addition, although the embryonic disk models presented within this article are applicable and imaginable in all nondorsally conjoined twins, they remain abstract renderings which are created by rationally reasoning backward to early embryogenesis. This starting point could be too simplistic; the multifold of complicated molecular mechanisms during human embryogenesis could impossibly be overseen within this simplified model. Furthermore, the propositions that nondorsally united twins originate from the duplication of axially located structures and the assumption that dorsally united twins originate through a process of secondary fusion are still a mere conceptual conjecture. However, this article tries to break the paradigm in the current pathogenetic models in the genesis of united twins and criticizes the general view that all types of (conjoined) twinning, irrespective of the applied explanatory model, are placed in one overarching receptacle. Progress in embryological understanding will never occur if oversimplified theories are reinforced by standard concepts being repeated over and over (Boklage, 2009; Hoekstra et al., 2008).
